# Selective Targeting of the Hedgehog Signaling Pathway by PBM Nanoparticles in Docetaxel-Resistant Prostate Cancer

**DOI:** 10.3390/cells9091976

**Published:** 2020-08-27

**Authors:** Santosh Kumar Singh, Jennifer B. Gordetsky, Sejong Bae, Edward P. Acosta, James W. Lillard, Rajesh Singh

**Affiliations:** 1 Department of Microbiology, Biochemistry and Immunology, Cancer Health Equity Institute, Morehouse School of Medicine, Atlanta, GA 30310, USA; sksingh@msm.edu (S.K.S.); jlillard@msm.edu (J.W.L.J.); 2 Departments of Pathology and Urology, Vanderbilt University Medical Center, Nashville, TN 37232, USA; Jennifer.b.gordetsky@vumc.org; 3 Division of Preventive Medicine, University of Alabama at Birmingham School of Medicine, Birmingham, AL 35205, USA; bsejong@uab.edu; 4 Department of Pharmacology and Toxicology, University of Alabama at Birmingham, Birmingham, AL 35294, USA; eacosta@uab.edu

**Keywords:** prostate cancer, thymoquinone, drug transporter, nanoparticles, aptamer

## Abstract

An abnormality in hedgehog (Hh) signaling has been implicated in the progression of prostate cancer (PCa) to a more aggressive and therapy-resistant disease. Our assessments of human PCa tissues have shown an overexpression of the Hh pathway molecules, glioma-associated oncogene homolog 1 (GLI-1), and sonic hedgehog (SHH). The effect of the natural compound thymoquinone (TQ) in controlling the expression of Hh signaling molecules in PCa was investigated in this study. We generated planetary ball-milled nanoparticles (PBM-NPs) made with a natural polysaccharide, containing TQ, and coated with an RNA aptamer, A10, which binds to prostate-specific membrane antigen (PSMA). We prepared docetaxel-resistant C4-2B-R and LNCaP-R cells with a high expression of Hh, showing the integration of drug resistance and Hh signaling. Compared to free TQ, A10-TQ-PBM-NPs were more effective in controlling the Hh pathway. Our findings reveal an effective treatment strategy to inhibit the Hh signaling pathway, thereby suppressing PCa progression.

## 1. Introduction

Among men, prostate cancer (PCa) is the most common non-cutaneous malignancy; in 2020, an estimated 33,300 men will die from metastatic PCa [1]. The progression of PCa depends on alterations in the tumor’s genome [2]. Challenges associated with the treatment of PCa include (a) the progressive differentiation of tumors from indolent to aggressive forms, (b) therapeutics involving sequencing for metastatic and drug-resistant PCa and (c) a failure to implement biomarker-driven treatment [3]. Various molecular and clinical factors, including alterations in genes such as *ERG*, *SPOP*, *MYC*, androgen receptor (*AR*), and *CHD1*, drive PCa progression. The loss of *p53* and/or other tumor suppressor genes, reduced capacity for DNA repair, the dysfunction of telomerase activity, and changes in the pathways that govern the growth of cells also mediate the progression of PCa [2,4,5,6,7].

The hedgehog (Hh) signaling pathway is noted for its regulation of many cellular processes, including cell growth, cell differentiation, patterning, embryonic development, and organogenesis. In adult tissues, Hh maintains the stemness of cells and is involved in tissue repair and regeneration [8]. In mammals, the Hh signaling pathway contains three genes: *sonic*, *Indian*, and *desert hedgehog*, which are involved in the patterning of various tissues and structures [9]. These secreted Hh molecules bind to patched (PTCH1 and PTCH2), followed by inhibition of the seven-transmembrane protein smoothened (SMO), and subsequently trigger glioma (GLI)-dependent transcription [9,10]. As these proteins and pathways are necessary for various cellular processes, abnormal losses or gains in Hh signaling are associated with defects in development and malformations [9]. Furthermore, abnormalities in Hh pathways cause tumors to develop, such as basal cell carcinoma, medulloblastoma, rhabdomyosarcoma, fibroma, and meningioma [11]. Moreover, the inappropriate expression of the Hh signaling pathway is implicated in tumorigenesis in various tissues and is present in advanced stages of PCa [12,13,14,15].

As synthetic chemotherapeutic agents may cause side effects in cancer treatment, there is currently a search for natural products that are less toxic. For thousands of years, drugs derived from natural sources have been used to treat various diseases; today, they are being promoted for the treatment of cancers [16,17,18]. Among the drugs available from plant sources, thymoquinone (TQ), which is derived from *Nigella sativa*, also known as black seed, is used for its antioxidative and suppressive activity against carcinogenesis, eicosanoid production, and membrane lipid peroxidation [19]. TQ has antiproliferative effects on various cancers, including PCa [19,20,21,22]. A previous study used plant-based compounds to inhibit the Hh activity in PCa [23]. These promising results show the need for natural compounds that are effective in treating cancers. Although TQ acts on cancers by several mechanisms, its effects on the Hh pathway have not been explored. As various cancers, including PCa, show elevated levels of Hh, there is a need to study the effects of TQ on the Hh pathway.

During cancer treatment, apart from surgery, radiation therapy, and hormonal therapy, chemotherapy is a common form of treatment in which drugs are given systemically to patients to control the proliferation of cancer cells [24]. The drugs, although generally potent, are often lacking in efficacy due to nonspecific targeting and/or poor drug delivery. To circumvent drug delivery limitations, nanoparticles (NPs) have been developed to advance disease detection, diagnosis, and the targeted delivery of drugs [25,26]. In addition to the progress of nanotechnology, aptamer-based NPs have been introduced for the targeted delivery of drugs to treat cancers, with the goal of lowering toxicity and improving efficacy [27].

In the present investigation, we evaluate the effect of TQ on the Hh pathway to control the proliferation/progression of PCa. To evaluate the advantages of aptamer-bound, TQ-based NPs compared to TQ with NPs alone, we use the A10 RNA aptamer, which binds to prostate-specific membrane antigen (PSMA), which is exclusively expressed on the membranes of PCa cells. Aptamer-bound TQ is effective in controlling the abnormal expression of drug resistance genes and Hh pathway molecules/proteins.

## 2. Materials and Methods

### 2.1. Materials and Reagents

TQ, soluble starch, dimethyl sulfoxide (DMSO), and 1,2-distearoyl-sn-glycero-3-phosphoethanolamine)-polyethylene glycol-N-hydroxysuccinimidyl ester (DSPE-PEG-NHS) were purchased from Fisher Scientific (Pittsburgh, PA, USA). Polyethylene glycol (PEG) and polycaprolactone (PCL) were purchased from Sigma (St. Louis, MO, USA). A10 (truncated A10-3.2) aptamers were synthesized by Aptagen, LLC (Jacobus, PA, USA).

### 2.2. Synthesis of TQ-Encapsulated PBM-NPs

The planetary ball-milled (PBM) nanoparticle formulation is described in our previous publication [28]. Briefly, 4% starch (*w*/*v*, FDA approved) was dissolved in phosphate buffer saline (PBS) by heating and cooling it to room temperature. Texas red and TQ were added with continuous stirring, as described in US Patent 8,231,907. Following the patent, TQ-encapsulated NPs were placed in a jar holding heat-absorbing zirconium oxide planetary milling balls that were rotated around a common axis. Particle size was controlled by varying the centrifugal force (revolutions/s (Ω)), the number and size of the zirconium oxide balls, and jar velocity.

### 2.3. Synthesis of Nanoparticle-Aptamer Conjugates

To engineer the planetary ball-milled PBM-NP and aptamer conjugation, DSPE-PEG-NHS (20 mg) was dissolved in a 1:1 ratio of DMSO/water, then NHS-activated particles were covalently linked to 200 µL of 2F-modified PSMA aptamer (A10-3.2) (GGGAGGACGAUGCGGAUCAGCCAUGUUUACGUCACUCCU-spacer-NH2, 1 µg/µL in nuclease-free water) and mixed overnight at 4 °C. A preparation of HEPES (4-(2-hydroxyethyl)-1-piperazineethanesulfonic acid)-buffered saline (pH 7.4), 1 mM MgCl2, 1 mM CaCl2, 0.01% I-block (Tropix Inc., Bedford, MA, USA, Fisher Scientific), and 0.05% Tween 20 was used to dissolve the aptamer. The A10-3.2 aptamer–NP conjugate was washed, suspended in PBS, filtered, lyophilized, and stored as a powder.

### 2.4. Engineering of Aptamer-Conjugated, TQ-Encapsulated PBM-NPs

Briefly, 10 mg of TQ–starch NPs, conjugated with or without A10, were dissolved in methylene chloride (1 mL) and then coated with 2% activated PCL and PEG. Following our previous publication [28], to activate PCL, dry dioxane (6 mL) and PCL (2 g) was mixed and heated in a water bath. N, N’-disuccinimidyl carbonate and pyridine were added, and the preparation was placed in a shaker for 6 h. The precipitate was collected by filtration with diethyl ether, dissolved in acetone, and left to become a dry powder. Finally, the NPs were stirred at 4 °C overnight, and the remaining solvent was removed by evaporation under reduced pressure. The PBM-NPs were washed with deionized water and lyophilized.

### 2.5. Characterization of PBM-NPs

Following our previous publication [28], the particle size was measured at pH 6.8 using a Malvern Zetasizer ZS instrument. The NPs were at a concentration of 0.1 mg/mL (5% mass, assuming a density of 1 g/cm^3^). For the determination of structural and physicochemical properties, NMR was employed. The PBM-NPs modified with PEG and PCL were dissolved in deuterated water. The 1H-NMR spectra were recorded on a Bruker 400 MHz spectrometer. Transmission electron microscopy (TEM), which shows a 2D image of the inner structure, was used to determine the particle size distribution. A preparation of A10-TQ-NP was sonicated, and 3 µL was applied to a glowing discharged copper grid and incubated for 1 min, followed by two washings with 20 µL of buffer/water (side blot, Whatman filter paper 4). The preparation was stained with 0.75% uranyl formate and air-dried. The sample was imaged with a Talos 120LC microscope operating at 120 kV.

### 2.6. Nanoparticle Tracking Analysis (NTA) of PBM-NPs

Nanoparticle tracking analysis (NTA) allows for an analysis of the size distribution and concentration of NPs ranging in size from 0.01–1 µM. NTA was employed by use of NanoSight LM10 with NTA2.3 (NanoSight Ltd., Panalytical Inc., Westborough, MA, USA). The NPs were placed for 10 min in an ultrasonic bath and then introduced into the Nano Sight flow cell. The conditions for measurements were as follows: temperature 21.0 +/− 0.5 °C; viscosity = 0.99 +/− 0.01 cP, frames per second = 25, and measurement time = 30 s.

### 2.7. Super Plasmon Resonance (SPR) Analysis of A10-Conjugated PBM-NPs

To determine the interaction between A10 aptamer-conjugated PBM-NP and PSMA, we examined the binding affinity using the OpenSPR instrument (Nicoya LifeScience, Kitchener, ON, Canada). Following the manufacturer instructions, first, the carboxyl sensor chip was loaded into the instrument, and pumped with the running buffer, PBS (pH 7.4). Next, amine coupling was performed with a flow rate of 20 µL/min. A ligand, the PSMA 40 µg/mL antibody (R&D Systems, Minneapolis, MN, USA), was diluted into activation buffer (provided in amine coupling kit) and immobilized on a sensor chip followed by running buffer. After a 4-min interaction, blocking and then blank buffer were injected. Furthermore, various concentrations (125 nM–500 nM) of analyte A10-PBM-NPs were injected on the immobilized sensor chip with a flow rate of 20 µL/min. The interaction was assessed by subtraction of the buffer blank generated by the flow cell, and the data sets were analyzed by one to one (1:1) binding fit models using TraceDrawer software.

### 2.8. Immunohistochemistry

Human tissue microarray slides containing 62 clinical cases diagnosed as normal (*n* = 3) or pathological stage II (*n* = 33), stage III (*n* = 15), or stage IV (*n* = 11) PCa were procured from US BIOMAX, Inc. (Derwood, MD, USA). The staining method followed our previous publication [29]. In brief, paraffin-embedded tissue sections were deparaffinized in xylene and rehydrated in a graded alcohol series (100, 95, and 70%, 5 min each) followed by antigen retrieval (accomplished by incubating tissue for 10 min at 92 °C in a retrieving buffer) to enhance epitope availability and blocking with H_2_O_2_ (3%). The slides were rinsed with PBS+ 0.05% Tween 20 (PBS-T) and re-blocked with 5% normal donkey serum (Jackson Immuno Research, West Grove, PA, USA) for 1 h at room temperature. The slides were incubated with an anti-sonic hedgehog (SHH) antibody (1:200, Novus Biologicals, USA) at 4 °C overnight or with anti-GLI1 antibody (1:300, Novus Biologicals, USA) for 4 h. The slides were washed with PBS-T and incubated with a donkey anti-rabbit secondary antibody for 1 h at room temperature (R&D Systems, Minneapolis, MN, USA). The slides were washed with PBS-T and incubated with streptavidin–alkaline phosphatase (Jackson Immuno Research) for SHH, and streptavidin-horseradish peroxidase (Biolegend, San Diego, CA, USA) for GLI1, and developed in alkaline phosphatase (AP) red chromogen coloring agent or diaminobenzidine (DAB), respectively. The slides were counterstained with hematoxylin (Fisher Scientific), and dehydrated. A 40× objective lens (Histowiz Inc., Brooklyn, NY, USA) captured digital images from the slides. The positive pixel counts for SHH and GLI1, in normal tissues and in adenocarcinomas of various stages, were quantified using algorithms provided by an Aperio Image Scope scanning system (Aperio Technologies, Vista, CA, USA).

### 2.9. Cell Lines and Generation of Docetaxel (DTX)-Resistant Cells

PCa cell lines, C4-2B and LNCaP, were purchased from American Tissue Culture Collection (ATCC, Manassas, VA, USA). C4-2B cells were maintained in DMEM plus F12 Medium (Fisher Scientific) supplemented with 10% heat-inactivated fetal bovine serum (FBS), insulin, triiodo-L-thyronine, transferrin, D-biotin, adenine (Millipore Sigma, St. Louis, MO, USA), and 10,000 U/mL penicillin/10,000 µg/mL streptomycin antibiotic solution (Fisher Scientific). LNCaP cells were maintained in RPMI-1640 media supplemented with 10% FBS, nonessential amino acids, HEPES, 2 mM L-glutamine, and penicillin/streptomycin antibiotic solution (Fisher Scientific). Docetaxel (DTX)-resistant cell lines were generated from parental C4-2B and LNCaP cells by gradually increasing the DTX concentration from 2 nM to 160 nM and were maintained in an incubator for 7 months at 37 °C and 5% CO_2_. Likewise, resistant LNCaP cells were generated by gradually increasing the DTX concentration from 2 nM to 60 nM. The DTX-resistant cells, C4-2B-R and LNCaP-R, were maintained in RPMI-1640 media supplemented with 10% FBS, nonessential amino acids, HEPES, 2 mM L-glutamine, and penicillin/streptomycin antibiotic solution (Fisher Scientific). Both cell lines were maintained in an incubator at 37 °C and with 5% CO_2_.

### 2.10. MTT Assay

To evaluate the therapeutic potentials of TQ and PBM-NPs conjugated with or without the A10 aptamer, cell viability assays were performed for C4-2B-R and LNCaP-R cells. Following our previous publication [28], growing cells were trypsinized (0.25% trypsin, Fisher Scientific). Cells (10,000/well) were seeded in 96-well plates for overnight incubation and then exposed to a sequential dosage of free TQ (10, 20, 40, 80, 160, 320 µM), and PBM-NPs (TQ-NP, or A10-TQ-NPs; 5, 10, 20, 40, 80, 100 µM) for 24, 48, or 72 h at 37 °C in a 5% CO_2_ incubator. Next, 3-(4,5-dimethylthiazol-2yl) 2,5-diphenyltetrazolium bromide (MTT, 5 mg/mL, Sigma, St. Louis, MO, USA) was added to each well, followed by incubation at 37 °C for 3–4 h. The purple formazan crystals were dissolved in 100 µL of DMSO, and the absorbance was measured at 570 nm with a spectrophotometer (Spectramax M5, Molecular Devices, San Jose, CA, USA). Furthermore, the IC_50_ values (half-maximum inhibitory concentration) were calculated for C4-2B-R and LNCaP-R cells. (IC_50_ values for free TQ were determined for both parental and DTX-resistant cells; IC_50_ values for PBM-NPs (TQ-NPs and A10-TQ-NPs) were calculated for DTX-resistant cells). Likewise, to ensure DTX tolerance in the cell lines generated, C4-2B-R and LNCaP-R cells were treated with DTX at varying concentrations (1, 10, 20, 50, 100, 200, 300, or 500 nm) for 24, 48, or 72 h at 37 °C in a 5% CO_2_ incubator, followed by an MTT assay.

### 2.11. Immunofluorescence Evaluations

Parental C4-2B and LNCaP and resistant C4-2B-R and LNCaP-R cell lines were seeded in 48-well plates overnight at 37 °C in a 5% CO_2_ incubator. Following our protocol [30], cells were washed with cold PBS, fixed in 4% paraformaldehyde (PFA), permeabilized with 0.01% saponin, and blocked with 3% bovine serum albumin for 30 min. The cells were treated with primary antibodies, anti-SHH (1:150) and anti-GLI1 (1:150) (Novus Biologicals, Littleton, CO, USA), overnight at 4 °C followed by a secondary Alexa Fluor 594 antibody (Cell Signaling Technology, Danvers, MA, USA) for 1 h at room temperature. Next, the cells were washed and stained with Phalloidin 488 green solution (1:50, Fisher Scientific) for 30 min to visualize the F-actin cytoskeleton. The nuclei were counterstained with 4′,6-diamidino-2-phenylindole (DAPI, Invitrogen, Carlsbad, CA, USA) and imaged with an EVOS FL microscope (Thermo Fisher Scientific, Carlsbad, CA, USA).

To assess the cellular uptake of NPs through the PSMA, C4-2B-R and LNCaP-R cells were grown overnight in 8-well slides (Fisher Scientific) and then treated with Texas red-conjugated empty-NPs or A10-TQ-NPs for 2 h. Moreover, cells were washed thrice with PBS, incubated with the primary antibody, anti-PSMA (8 µg/mL, R&D Systems, Minneapolis, MN, USA) overnight at 4 °C, followed by exposure to secondary goat anti-mouse Alexa Fluor 488 (Invitrogen, Carlsbad, CA, USA) for 1 h. Nuclei were counterstained with DAPI and imaged with a fluorescent microscope.

### 2.12. Flow Cytometry Assay

C4-2B-R and LNCaP-R cells were grown in six-well plates overnight and treated with empty NPs or TQ-NPs conjugated with or without the A10 aptamer for 48 h. Following our previous publication [30], the cells were washed with FACS buffer (2% FBS), harvested with Accutase, and counted with a hemocytometer (Countess II FL, Life Technology, Carlsbad, CA, USA). To assess the expression of PSMA, SHH, and GLI1, cells were fixed with 4% PFA, exposed to human Fc Block (BD Biosciences, San Jose, CA, USA), and stained with the primary antibodies for PSMA (2 µg/mL, R&D Systems, Minneapolis, MN, USA), SHH (1:100, Novus Biologicals, Littleton, CO, USA), and GLI1 (1:100, Novus Biologicals) overnight at 4 °C followed by an fluorescein isothiocyanate (FITC)-conjugated secondary antibody (for PSMA: Invitrogen, Carlsbad, CA, USA; for SHH and GLI1: Cell Signaling Technology, Danvers, MA, USA) at room temperature. Cells were washed twice and suspended in the FACS buffer. A Guava easyCyte HT (EMD Millipore, Billerica, MA, USA) flow cytometry instrument was used to analyze the data.

### 2.13. Quantitative Reverse Transcription PCR (qRT-PCR)

Following our previous publication [28], total RNA was extracted by the Trizol method (Invitrogen, Paisley, UK). C4-2B-R and LNCaP-R cells were treated with various concentrations of TQ-NP or A10-TQ-NP for 48 h and lysed with the Trizol reagent, followed by the isolation of mRNA. Next, 1 µg of mRNA was reverse-transcribed into a cDNA template using the RT-qPCR reagent kit according to the manufacturer’s instructions (Biorad, Hercules, CA, USA). The SYBR^®^ Green PCR master mix reagents (Biorad) and CFX-manager software (CFX96 Real-Time System, Biorad) were used to measure gene expression. The primers for the ATP-binding cassette (ABC) transporter and the Hh pathway listed in Table 1 were synthesized from the National Center for Biotechnology Information GeneBank database.

### 2.14. Western Blot Analyses

DTX-resistant PCa cells were grown overnight and treated with TQ-NP or A10-TQ-NP for 48 h. The method for protein isolation followed our previous publication [28]. Radioimmunoprecipitation assay (RIPA) buffer containing 1Xprotease and phosphatase inhibitor cocktail (Thermo Scientific, Rockford, IL, USA) was added to lyse the cells. The protein concentrations were determined with bicinchoninic acid protein assay kits (Thermo Scientific, Rockford, IL, USA). To compare drug resistance between parent and DTX-resistant cells, we isolated the proteins from C4-2B and LNCaP cells. Protein samples were denatured by adding SDS-PAGE 1XLamelli buffer and heating to 95 °C for 10 min. Samples of protein (30 µg) were loaded on 4–12% precast gels (Life Technologies, Carlsbad, CA, USA). After electrophoresis, the proteins were transferred to polyvinylidene fluoride (PVDF) membranes. The immunoblots were blocked in 5% non-fat dry milk (Biorad) for 1 h, as previously described [28]. For the detection of proteins, immunoblots were probed with the primary antibodies anti-Abcb1, -SHH, -GLI1, -PTCH1, and -suppressor of fused (SUFU) (1:1000, Cell Signaling Technology, Danvers, MA, USA) and -PSMA (1 µg/mL, R&D systems, Minneapolis, MN, USA) overnight at 4 °C followed by secondary antibodies, anti-mouse and/or anti-rabbit (1:2000), for 2 h at room temperature. Subsequently, they were washed with PBS-T three times. GAPDH (1:1000, Cell Signaling Technology, Danvers, MA, USA) was used as an internal control to ensure equal loading. The immunoblots were developed with Western blotting chemiluminescent detection reagent (GE Healthcare-Biosciences, Pittsburgh, PA, USA), and images were captured by ImageQuant LAS4000 (GE Healthcare-Biosciences, Pittsburgh, PA, USA). Image-J software (NIH) was used to quantify the band intensities of proteins.

### 2.15. Statistical Analysis

Experimental data were presented as means and standard errors of means (±SEM) for at least three independent experiments. An analysis of variance followed by Tukey’s post-hoc test or pre-defined contrast and a two-sample *t*-test were applied for comparison across groups and between groups, respectively. Additionally, Student’s paired *t*-test was applied for comparisons within groups. The intensities of SHH and GLI1 were tested for normality assumptions using the Shapiro–Wilk test. Stat view II programs (Abacus Concepts, Inc., Berkeley, CA, USA) were used to analyze the data and were considered statistically significant if *p* values were <0.05. With FlowJo Software, the Kolmogorov–Smirnov two-sample test was used to compare the distribution of SHH and GLI1.

## 3. Results

### 3.1. Characterization of the PSMA Aptamer (A10)-Conjugated, TQ-Encapsulated PBM-NPs

**Figure 1:**
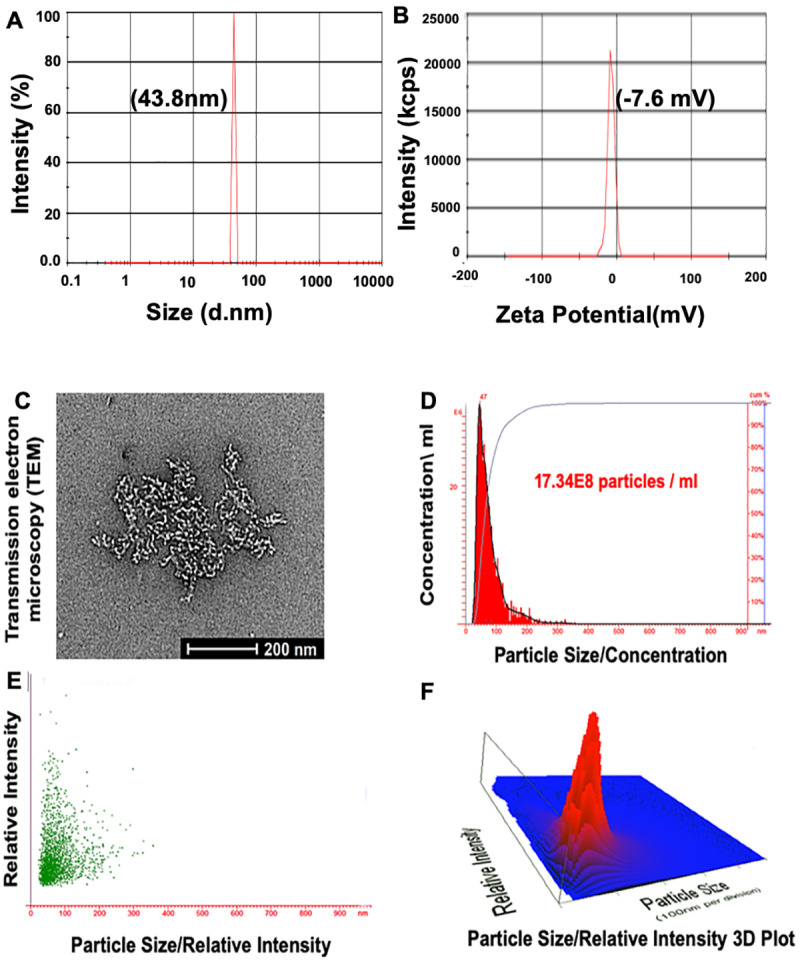
Characterization of prostate-specific membrane antigen (PSMA) aptamer (A10)-conjugated, thymoquinone (TQ)-encapsulated planetary ball-milled nanoparticles (PBM-NPs). (**A**) Particle size distribution measured at pH 6.8, and (**B**) zeta potential measured by a Malvern Zeta Sizer instrument. (**C**) TEM showing a 2D micrograph of PBM-NPs, and (**D**–**F**) the particle size/concentration, particle size/relative intensity, and a particle size/relative intensity 3D plot for aptamer-conjugated PBM-NPs, determined by use of a NanoSight LM10 instrument.

For the development of NPs, the characterization of the particles is essential. Formulations of NPs should be characterized by size, charge, and biophysical and chemical properties. To do so, the following criteria were applied: (1) a biocompatible and biodegradable nanocarrier, (2) efficient binding to the negatively charged aptamer, (3) high cellular uptake, and (4) extended blood circulation times. The zeta size of PBM-NPs (A10-conjugated-TQ-NPs) was 43.8 nm (Figure 1A). This result is consistent with reports demonstrating that, for NPs, sizes of <50 nm are most suitable for intracellular uptake [28]. The zeta potential was −7.6 mV (Figure 1B), which minimizes nonspecific interactions with the negatively charged A10 aptamer. In addition, negatively charged NPs have a strong binding capacity to the positively charged molecule/or membrane (PSMA^+^). Moreover, the NP surface is coated with PEG and PCL, which decreases non-specific binding to the cell membrane and allows for longer circulation times. Furthermore, PEG modification on NP surfaces affects their stability and maximizes the blood circulation half-life [31]. TEM was employed to determine the size, size distribution, and morphology of NPs. The A10-TQ-NPs were spherical, regardless of the conditions. A TEM micrograph of the fabricated NPs is shown in Figure 1C. Spherical NPs allow more cellular uptake compared to other NPs, for example, those that are needle-shaped [28].

To determine the size distribution of PBM-NPs per mL of solution, we employed nanoparticle tracking analysis (NTA). NTA, used for the measurement of NPs, carbon nanotubes, and viral particles [32], utilizes the Brownian motion of particles and light scattering to measure the size distribution and concentration of particles in a liquid solution. By NTA, values for particle size and concentration were 47 nM and 17.34 × 10^8^ particles/mL of solution, respectively. Figure 1D–F show the particle size/concentration, particle size/relative intensity, and a particle size/relative intensity 3D plot. In summary, the size, zeta potential, TEM, and NTA results demonstrate that A10-TQ-NPs have a small size, a spherical shape, a high sensitivity to the positively charged membrane, a uniform size distribution, and a high concentration.

### 3.2. NMR Characterization, SPR Binding Affinity, and Cellular Uptake of TQ-Encapsulated PBM-NPs

**Figure 2:**
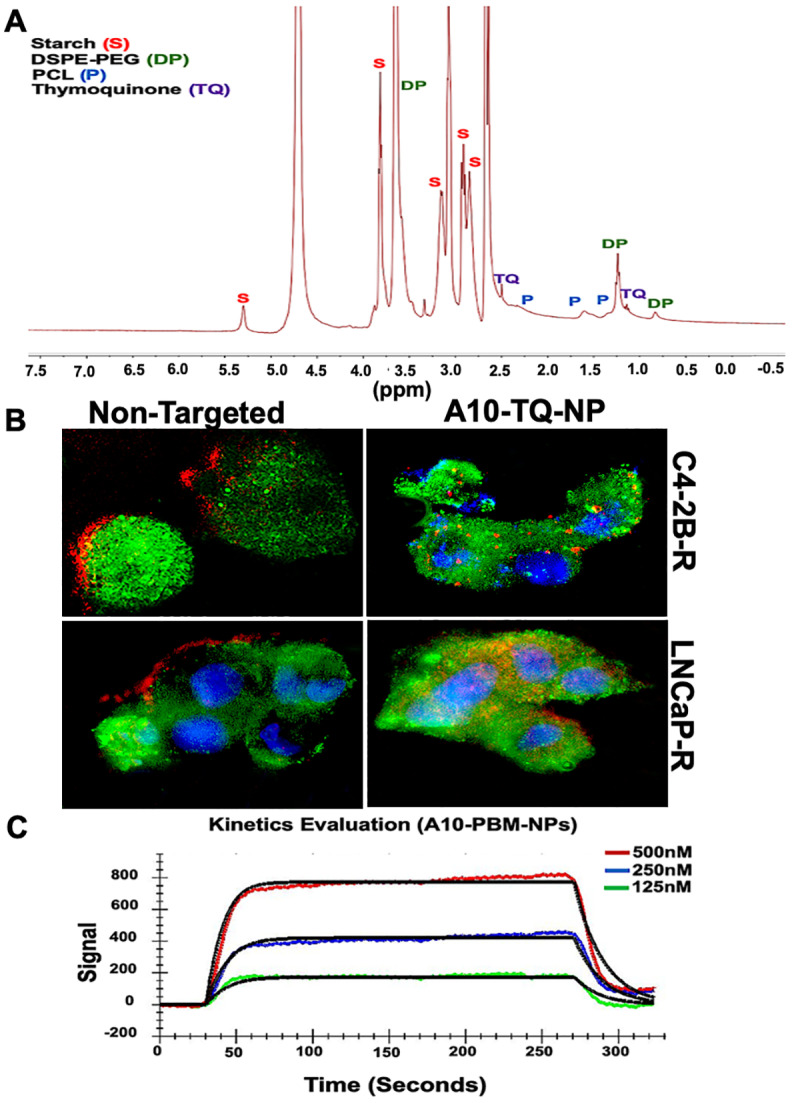
1H-NMR spectrum and cellular internalization of TQ-encapsulated PBM-NPs. (**A**) 1H-NMR spectra of starch, TQ, polyethylene glycol (PEG) and polycaprolactone (PCL), showing the conjugation of TQ-encapsulated-PBM-NPs. The 1H-NMR spectra were recorded on a Bruker 400 MHz spectrometer. (**B**) Cellular internalization of A10 aptamer-conjugated TQ-PBM-NPs in drug-resistant prostate cancer (PCa) cells (C4-2B-R and LNCaP-R) compared to non-targeting, empty PBM-NPs. Red: Texas red conjugated PBM-NPs; green: PSMA stained with FITC-conjugated antibody; blue: nuclei counterstained with 4′,6-diamidino-2-phenylindole (DAPI). Scale bar, 50 µM. (**C**) Surface Plasmon Resonance (SPR) analysis of binding interaction between the PSMA and A10 aptamer-conjugated PBM-NPs. A typical response curve from experimental data at different analyte concentrations (green, 125 nM; blue, 250 nM; red, 500 nM) shown the association (ka = 1.36 × 10^5^) and dissociation (kd = 8.18 × 10^−2^) phase of A10-PBM-NPs and PSMA interactions. A kinetic analysis was performed using globally fitted to 1:1 binding model represented by black lines. Data were analyzed using the TraceDrawer evaluation software.

NMR spectroscopy has a wide range of applications in the field of biomedical engineering and material science, including NP engineering. It is valuable when NPs are produced in different sizes, and when surface chemistry and size are key factors in determining the characteristics of NPs [33]. Although surface chemistry can be characterized by the use of FTIR spectroscopy and Raman spectroscopy, NMR provides information related to structure and surface chemistry. Here, we applied NMR to characterize A10-TQ-encapsulated-NPs for these factors. Nuclear magnetic resonance (NMR) spectra of 13C analyses showed the conjugation of TQ, starch, PEG, and PCL in A10 fabricated PBM-NPs. The peak at 3.6 ppm showed the presence of PEG; peaks at 0.8 and 1.2 indicated the presence of DSPE; peaks at 5.3 (anomeric), 3.8, 3.0, and 3.1 ppm showed the presence of starch (Figure 2A). Peaks at 1.5, 1.6, and 2.3 showed the presence of PCL, and the peaks at 1.4 and 2.5 showed the presence of TQ. These results demonstrate the intended surface modifications of PCL-PEG on TQ-PBM-NPs.

The cellular uptake of NPs depends on their size, shape, surface charge, hydrophobicity, and surface functionality. Tumor cell imaging is the most appropriate technology to use in optimizing cellular targeting, uptake, and trafficking [34]. To investigate the cellular uptake and/or efflux of NPs, DTX-resistant PCa cells were treated with A10-conjugated TQ-NPs or empty NPs (non-targeted) for 2 h. C4-2B-R cells were treated with A10-TQ-NPs (22 µM); LNCaP-R cells were treated with A10-TQ-NPs (31 µM). Both were then evaluated by immunofluorescence. A10 aptamer-conjugated PBM-NPs were internalized by cells via receptor-mediated endocytosis; non-targeted (non-conjugated-PBM) NPs were bound to the outer surfaces of the cells (Figure 2B). Thus, the PBM-NPs are equipped with a homing device (the PSMA aptamer) that guides the PBM-NPs to the target site, the PSMA receptor. The results suggest that, within the cells, a PBM-NP-interaction could counteract the effect of multidrug resistance (MDR) efflux.

Targeted delivery at the disease site is currently of great interest in the biomedical field. From the recent works on biosensors, Surface Plasmon Resonance (SPR) has been widely used to monitor the biomolecular interactions that can detect the interaction between functionalized nanoparticles with a specific ligand [35,36]. To investigate the binding affinity between A10-PBM-NP and PSMA, we employed covalent coupling on sensor ship-based SPR. A typical response curve from experimental data at different analyte concentrations is shown in Figure 2C. Our results show that PSMA binds to A10-PBM-NP (the analyte) with a high association rate constant (ka = 1.36 × 10^5^ M^−1^S^−1^) and dissociation rate constant (kd = 8.18 × 10^−2^ S^−1^). The analyzed binding kinetics showed an equilibrium constant (KD) value of 6.02 × 10^−7^ M. Altogether, A10-PBM-NP displayed a strong interaction with the PSMA receptor.

### 3.3. PCa Cancer Tissues Display Altered Expressions of SHH and GLI1 that Increase with Higher Cancer Stages

**Figure 3:**
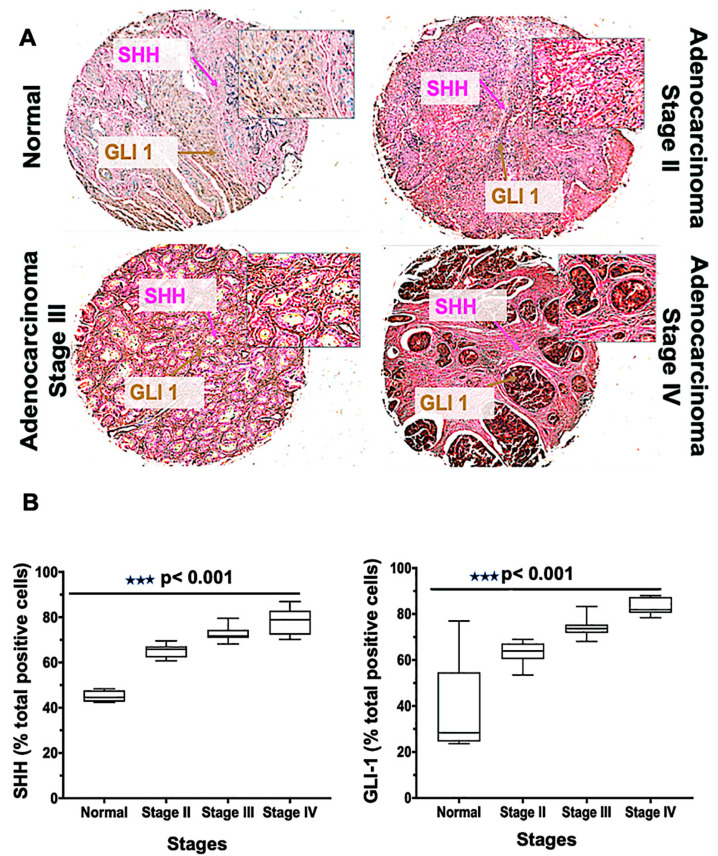
Glioma-associated oncogene homolog 1 (GLI-1), and sonic hedgehog (SHH) expression in human prostate tissues. (**A**) Tissues from normal prostate and adenocarcinoma stages II, III, and IV were stained with anti-SHH and anti-GLI1 antibodies. Representative cases showing immuno-intensities of SHH (Alkaline phosphatase (AP), magenta) and GLI-1 (diaminobenzidine (DAB), brown). An Aperio ScanScope Scanning system with a 40× objective lens captured digital images from the slide. Stained cells were categorized as to stain intensity 0 (blue), 1 + (yellow), 2 + (orange), and 3 + (red). There are differences (*p*  <  0.001) between the normal and adenocarcinoma groups. (**B**) Box plots showing the percentages of cells positive for SHH and GLI1 from each diagnosis group.

Tissue microarrays [19] are a high-throughput technology that permit the rapid assessment of biomarkers in tumor tissues. The Hh family has been implicated in tumorigenesis for men with mCRPC, and its level of activity correlates with the severity of the tumors [37]. To define the role of Hh signaling molecules, we performed SHH and GLI1 staining of a TMA consisting of 62 cases (three normal tissues, and 59 various clinical stages) of PCa. The expressions of SHH and GLI1 were higher in adenocarcinoma clinical stages III and IV compared to stage II; in normal tissue, their expressions were minimal (Figure 3A). A similar observation was seen in the meta-analysis of tissue data, in which seven out of 59 tumors were negative for SHH but positive for other proteins [10], indicating activation of Hh by downstream protein expression. In addition, there were more cells expressing SHH and GLI1 for PCas of stage IV and stage III compared to those of other samples (*p* < 0.001) (Figure 3B). The average median values of cells positive for SHH were 79, 73, 65, and 45 in stages IV, III, II, and normal, respectively. For GLI1, the values were 84, 74, 64, and 37, respectively. Thus, the expression of SHH and GLI1 increased with the adenocarcinoma stages of cancer, suggesting a role of the Hh pathway in PCa proliferation.

### 3.4. PSMA Aptamer (A10)-Conjugated, TQ-Encapsulated PBM-NPs Sensitize DTX-R PCa Cells to Low Concentrations of TQ

To define efficacious concentrations of TQ-PBM-NP and A10-TQ-PBM-NP, the viability of both resistant cell lines was tested at three time intervals and compared with that for free TQ. For cells treated for 48 h, PBM-NPs caused appreciable cytotoxicity. For free TQ, the IC_50_ values for C4-2B-R and LNCaP-R cells were 139 μM and 159 μM, respectively (Supplementary Figure S1). However, the IC_50_ values of A10-TQ-NP were 22 μM and 31 μM in C4-2B-R and LNCaP-R cells, respectively, which were approximately 6.3-fold and 5.1-fold lower than free TQ. With TQ-NP treatment, C4-2B-R and LNCaP-R cells had IC_50_ values of 73 μM and 87 μM, respectively (Supplementary Figure S1). These results show that, for resistant cells, the A10 conjugated-PBM-NP IC_50_ values were nearly three-fold lower compared to non-conjugated PBM-NP. These results indicate that, for both types of resistant cells, IC_50_ values were concentration dependent.

### 3.5. Drug-Resistant PCa Cells Overexpress ATP-Binding Cassette (ABC) Transporter and Hh Pathway Genes

**Figure 4:**
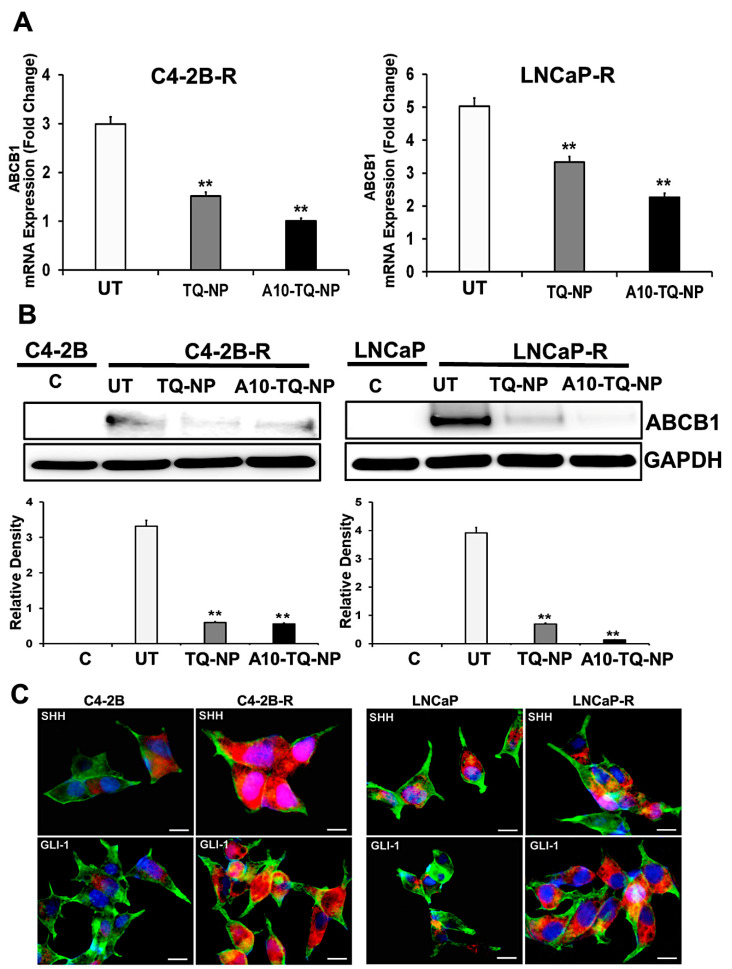
Gene expression changes in the ATP-binding cassette (ABC) transporter and the hedgehog (Hh) pathway in drug-resistant PCa cells. (**A**) Docetaxel (DTX)-resistant (C4-2B-R and LNCaP-R) PCa cells were treated with or without A10-conjugated TQ-NPs for 48 h. The expression levels of *ABCB1* were measured by RT-PCR and expressed as the fold change relative to the controls (parental cells, C4-2B or LNCaP); 18S rRNA was used as an internal control for data normalization. The experiments were repeated thrice. The data, presented as means and standard errors of means (±SEM), were analyzed by Student’s *t*-test. The asterisks ** indicate *p* values ≤ 0.01. UT: untreated (DTX-resistant) (**B**) DTX-resistant PCa cells were treated with or without A10-conjugated TQ-NPs for 48 h, and ABCB1 protein expression was assessed by Western blots. GAPDH was used as an internal standard for equalizing the samples. The lower panel shows the densitometry of corresponding Western blots. UT: untreated (DTX-resistant); C: control (parent cells). The experiments were repeated thrice. The data are presented as means ± SEM; the asterisks ** indicate *p* values ≤ 0.01. (**C**) Immunofluorescence expression of SHH and GLI1 markers in PCa cells. The parental (C4-2B and LNCaP) and DTX-resistant (C4-2B-R and LNCaP-R) PCa cell lines were stained with anti-SHH and GLI-1 antibodies. Images were captured by an EVOS-FL fluorescent microscope with a 40× objective lens and with an appropriate filter. Red: SHH and GLI; green: F-actin cytoskeleton stained with Phalloidin 488; blue: nuclei counterstained with DAPI. Scale bar, 50 µM.

In clinical settings, cancer cells resistant to chemotherapeutic drugs through MDR involving ABC efflux proteins is a challenge. Activation of the Hh pathway regulates MDR, in part, through ABC transporters [38]. In this context, we investigated whether drug resistance affected the cellular expression of molecules in the Hh pathway. First, we developed DTX-resistant C4-2B and LNCaP cells by exposure to DTX and examined the ABCB1 gene expression markers by RT-PCR. There were three- and five-fold elevated expressions of ABCB1 (MDR1) in C4-2B-R and LNCaP-R cells, respectively (Figure 4A). For cells treated with TQ-NPs or A10-TQ-NPs, there were low levels of ABCB1 RNA (Figure 4A). In addition, Western blots showed that the expression of ABCB1 was high in resistant cells compared to C4-2B and LNCaP parental cells (Figure 4B). This was corroborated by RT-PCR studies showing that TQ-NPs caused low levels of the ABCB1 protein. In cells treated with A10-TQ-NPs, there was significant downregulation of ABCB1. Next, to investigate whether drug resistance affects the cellular expression of Hh, we evaluated the parental and drug-resistant PCa cells by the use of SHH and GLI1 antibodies. Immunofluorescence studies showed high expressions of SHH and GLI1in LNCaP-R and C4-2B-R cells compared to parent cell lines, C4-2B and LNCaP (Figure 4C). These results suggest that the Hh pathway and drug resistance are linked.

### 3.6. A10-Conjugated, TQ-Encapsulated PBM-NPs Downregulate PSMA and Hh Proteins in DTX-Resistant PCa Cells

**Figure 5:**
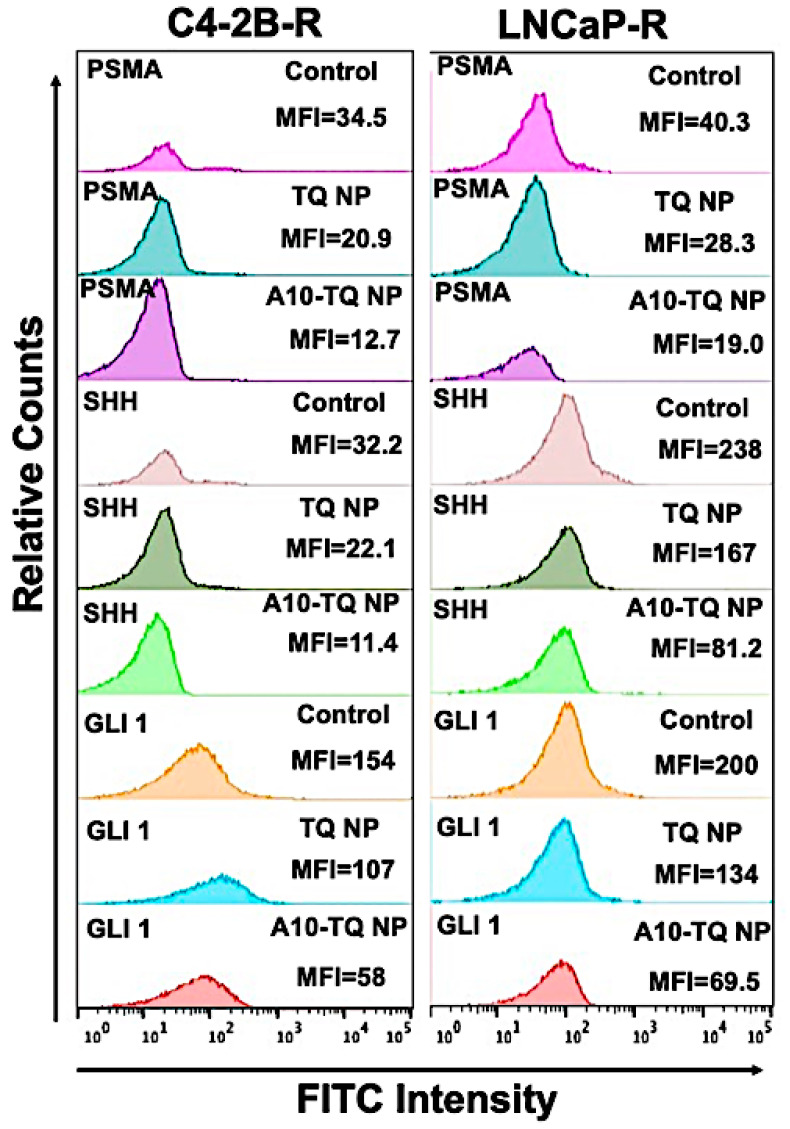
Relative mean fluorescence intensity of PCa cells treated with or without A10 aptamer-conjugated TQ-NPs. DTX-resistant PCa cells were treated with TQ-NP or A10-TQ-NP for 48 h and stained with FITC-conjugated antibodies. Relative counts were measured for PSMA, SHH, and GLI1 makers and analyzed by FlowJo software.

To determine the effect of A10-TQ-NPs and TQ-NPs in downregulating Hh proteins and PSMA, mean fluorescent intensities (MFIs) were evaluated in DTX-resistant cells treated with TQ-NP or A10-TQ-NP. The relative counts were measured by flow cytometry for cells stained with FITC-conjugated PSMA, SHH, and GLI1 antibodies. The MFIs of PSMA expression in C4-2B-R and LNCaP-R cells treated with A10-TQ-NPs were 12.7 and 19.0 compared to TQ-PBM-NP (20.9 and 28.3) and control cells (34.5 and 40.3), respectively. These data corresponded to the MFIs of SHH and GLI1 where the expressions of SHH (C4-2B-R: 11.4; LNCaP-R: 81.2) and GLI1 (C4-2B-R; 58; LNCaP-R: 69.5) were low upon treatment with A10-TQ-NP compared to values for TQ-NP and control cells (Figure 5). The data suggest that drug-resistant cells have high Hh signaling and that targeting this through PSMA aptamer-conjugated NPs inhibits the pathway and would increase the response of cancer cells to therapeutics.

**Figure 6:**
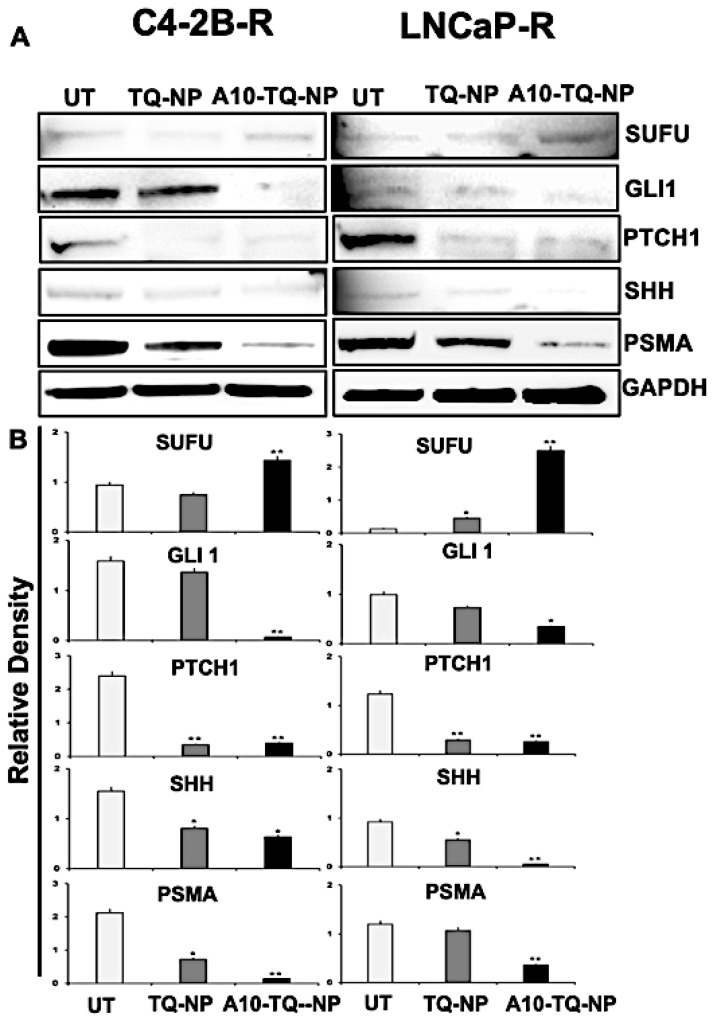
Immunoblot detection of Hh signaling proteins and PSMA expression in drug-resistant PCa cells targeted with NPs. (**A**) DTX-resistant (C4-2B-R and LNCaP-R) PCa cells were treated with or without A10-conjugated TQ-NP for 48 h, and the expression of PSMA, SHH, PTCH1, GLI1, and SUFU proteins were analyzed by Western blots. GAPDH was used as a loading control. Untreated (UT; DTX resistant). (**B**) The lower panel shows the densitometry of corresponding immunoblots. The data, presented as means ± SEM, were analyzed by Student’s *t*-test. The asterisks * and ** indicate *p* values ≤ 0.05 and < 0.01, respectively.

To evaluate the effect of TQ-NPs conjugated with or without the A10 aptamer on proteins, C42B-R and LNCaP-R cells were treated for 48 h with NPs (C4-2B-R, A10-TQ-NP, 22 µM and TQ-NP, 73 µM); (LNCaP-R, A10-TQ-PBM-NP, 31 µM and TQ-NP, 87 µM). Upon treatment of cells with A10-TQ-NPs, expressions of SHH, PTCH1, and GLI1 were low compared to controls (Figure 6A,B). In addition, SUFU, a negative regulator of the SHH pathway, was elevated in cells treated with A10-TQ-NP. PSMA expression was lower in cells treated with the A10 aptamer-conjugated NPs compared to non-conjugated TQ-NPs. Thus, in PCa cells, the A10 aptamer-conjugated PBM-NPs block the Hh pathway. As such, targeting the Hh pathway in this manner could suppress the progression of PCas.

### 3.7. A10-Conjugated, TQ-Encapsulated PBM-NPs Alter mRNA Expression of Hh Signaling Molecules in DTX-Resistant PCa Cells

**Figure 7:**
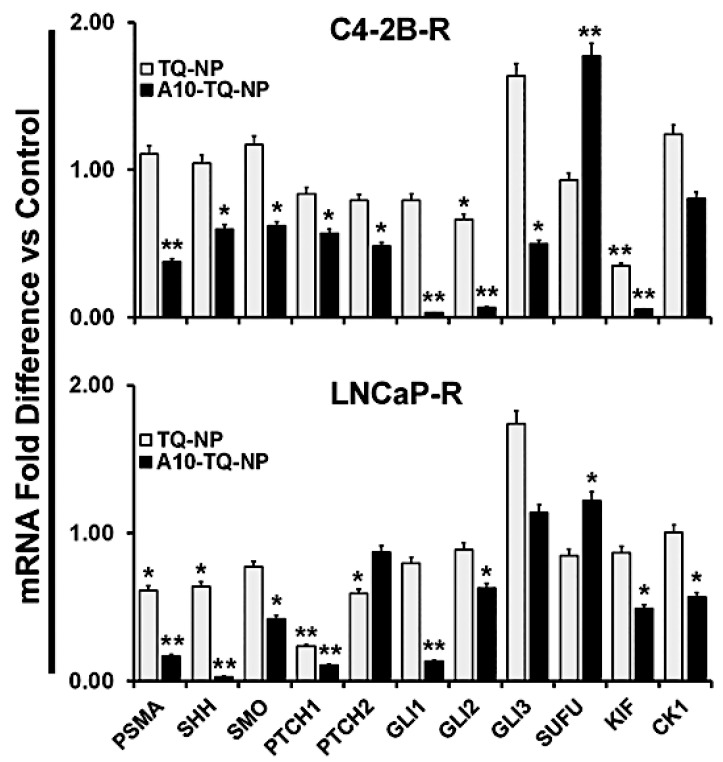
Validation of Hh signaling and PSMA at the mRNA level in DTX-resistant PCa cells. DTX-resistant (C4-2B-R and LNCaP-R) PCa cells were treated with or without A10-conjugated TQ-NP for 48 h. Levels of the indicated genes were measured in fold change relative to control cells by using RT-PCR. 18S rRNA was used as an internal control for data normalization. The experiments were repeated thrice. The data, presented as means ± SEM, were analyzed by Student’s *t*-test. The asterisks * and ** indicate *p*-values ≤ 0.05 and 0.01, respectively.

To confirm the effects on Hh signaling, we investigated the mRNA expression of Hh signaling molecules in DTX-resistant cells (C42B-R and LNCaP-R) treated with or without A10-conjugated TQ-NPs. Like the findings for protein expression, mRNA expression of these molecules was down-regulated in both types of cells treated with A10-TQ-NP (Figure 7). In C4-2B-R cells, the expression of SUFU was higher compared to that in cells treated with TQ-NPs. Upon treatment of cells with A10-conjugated NPs, SHH expression was lower in both cell lines compared to treatment with TQ-NPs. Moreover, a reduction in the Hh signaling cascade (GLI1, GLI2, KIF, CK1, SMO, PTCH1, and PTCH2) was evident for cells treated with A10-TQ-NPs. Thus, PCa is Hh dependent, and inhibiting Hh with NPs could provide a new avenue for therapy and improved quality of life for patients.

## 4. Discussion

The Hh signaling pathway is necessary for the growth, survival, and organization of various cells, tissues, and organs in the body [39]. Dysregulation of the Hh pathway is implicated in the development of various cancers, including skin carcinomas, medulloblastomas, pancreatic cancers, and lung cancers [11]. PTCH and SMO proteins are gatekeepers of the Hh pathway. Loss of function of PTCH, mainly due to mutations in the gene encoding this protein [40,41], causes elevated levels of GLI1 [42]. Similarly, with mounting evidence that the Hh pathway is involved in PCa, elevated levels of SHH and GLI1 were found in PCa cells, suggesting the possibility of their role as markers for abnormalities in the Hh pathway in PCa [43]. SHH signaling leads to activation of the transcriptional factors, GLI1, GLI2, and GLI3; GLI1 is a readout in the SHH pathway [44]. In the present study, the staining of SHH and GLI1 in PCa tissue samples showed similar results, signifying the role of Hh in PCa proliferation. Our findings are supported by the work of others showing that GLI1 expression is a crucial marker of the Hh pathway [14,45].

As there are many pathways that lead to the aberrant expression of Hh pathway proteins, SHH protein expression is unlikely to be the primary trigger for the Hh pathway, although previous studies have shown its expression in PCa [10,14,45,46]. Our observations, made with clinical samples of PCa, were in concordance with the concept that higher expressions of GLI1 and SHH are associated with higher stages of PCa. There are similar observations in studies of mouse xenografts wherein the overexpression of SHH leads to higher tumor grades [46]. In vivo and in vitro studies conducted on the SHH antibody-mediated suppression of PCa provide evidence for autocrine Hh signaling in PCa [45]; this is also supported by another study [47]. Although our work does not demonstrate autocrine/paracrine Hh signaling in PCa, we show that expressions of SHH and GLI are markers for identifying PCa for treatment.

Several features are common among cancer cells and stem cells. One such feature is chemoresistance. As cancer cells acquire the stem cell nature, they express high levels of ABC proteins that efflux drugs out of the cell during treatment [48,49]. The Hh pathway promotes stemness characteristics of cancer cells, in part by inducing chemoresistance through regulating the transcription of ABC transporter genes [38]. The inhibition of the Hh pathway by cyclopamine and exposing cancer cells to various chemotherapeutic drugs increases the killing effect [38]. In contrast, prior exposure of cells to SHH ligands leads to resistance to drugs by inducing the expression of ABC transporter transcripts [38]. For various cancers, including PCa, these results show a positive correlation between the Hh pathway and chemoresistance [38,50]. In the present study, we developed drug-resistant PCa cell lines by continuously exposing them to DTX. For resistant cells, expression of the ABC transporter and Hh pathway proteins were elevated. The continuous exposure of cancer cells to drugs leads to the induction of efflux proteins, rendering them resistant [28], and the exposure of PCa cells to drugs leads to the increased expression of Hh proteins [38]. The Hh pathway induces chemoresistance, and the pressure exerted by chemotherapeutic drugs elevates Hh pathway proteins along with ABC transporter proteins. Further studies need to be conducted to determine the interdependent nature of drug resistance and the Hh pathway in cancer progression.

Hh is involved in the progression of PCa to a more advanced, castration-resistant disease [12,44,49]. Although various chemicals have an antagonistic effect on the Hh pathway proteins or cancer in general, environmental factors can reduce the risk of cancer. Since PCa develops later in life, natural compounds with activity against PCa can delay disease occurrence and have a positive effect on the quality of life [23]. We previously used TQ along with DTX to reduce the cytotoxic effects of the drug, and showed that, for PCa cells, DTX and TQ in combination had a greater inhibitory effect on the survival pathways compared to DTX alone [51]. In the present study, we assessed the inhibitory effect of TQ on the Hh pathway, which can, in turn, reduce the proliferation and the chemoresistance of cancer cells. Compared to our previous study [51], in which we used the free form of TQ, here, we used TQ bound to NPs alone or in combination with the aptamer A10. As the targeted uptake of NPs is used for the therapy of diseases in a specific manner, we used an A10 RNA aptamer that binds specifically to PSMA, which is expressed on cell surfaces of PCa. To demonstrate the study, we screened four PCa cell lines (DU145, PC3, C4-2B, and LNCaP) for the expression of PSMA^+^ cells (Supplementary Figure S4). In the present investigation, the A10-conjugated TQ bound to NPs was effective, even at low concentrations of TQ compared to TQ bound to NPs alone.

Activation of the SHH pathway leads to functional GLI proteins [43]. Target genes of the GLI pathway include cell cycle regulators, anti-apoptotic and angiogenic molecules, epithelial–mesenchymal transition regulators, cell fate and cell renewal molecules, as well as effectors of other developmental signaling pathways [49,52]. As targeting the GLI pathway in PCa reduces the progression of disease, aptamer-based, TQ-bound NPs offer a potentially safer alternative to chemical Hh inhibitors. In addition, A10-bound TQ was more effective in reducing the expression of GIL1. A regulator of GLI activity is SUFU, which binds to all three GLIs and controls the expression of downstream proteins, thereby arresting cell proliferation and affecting other properties of cancer cells [53,54]. This is supported by the fact that loss-of-function mutations in the SUFU gene are the only Hh aberrations found in PCa [10,37]. Although the pathways by which it acts are unknown, TQ elevated the expression of SUFU in PCa cell lines. In addition to the regulation of GLI and SUFU, TQ regulated other proteins in the Hh pathway, showing that it is a regulator of this pathway in PCa.

In cancer treatment, the efflux of chemotherapeutic drugs by ABC multidrug transporters is a mechanism responsible for the resistance of various cancers [55]. Overexpression of the ABC class of proteins occurs due to the increased abundance of the MDR1 genes, which can be induced by mutations, activation of Raf, anti-cancer drugs, and DNA-damaging agents [55]. Although TQ does not directly prevent the overexpression of efflux proteins, it can indirectly control their expression by inhibiting Raf activation or by regulating the expression of MAPK, which downregulates expression of the *MDR1* gene [56,57]. The mechanism by which TQ downregulates the *ABCB1* gene is not known. Since drug resistance and the Hh pathway are linked in PCa, TQ could control the Hh pathway by regulating the ABC efflux proteins or vice versa. However, this point needs further investigation.

**Figure 8:**
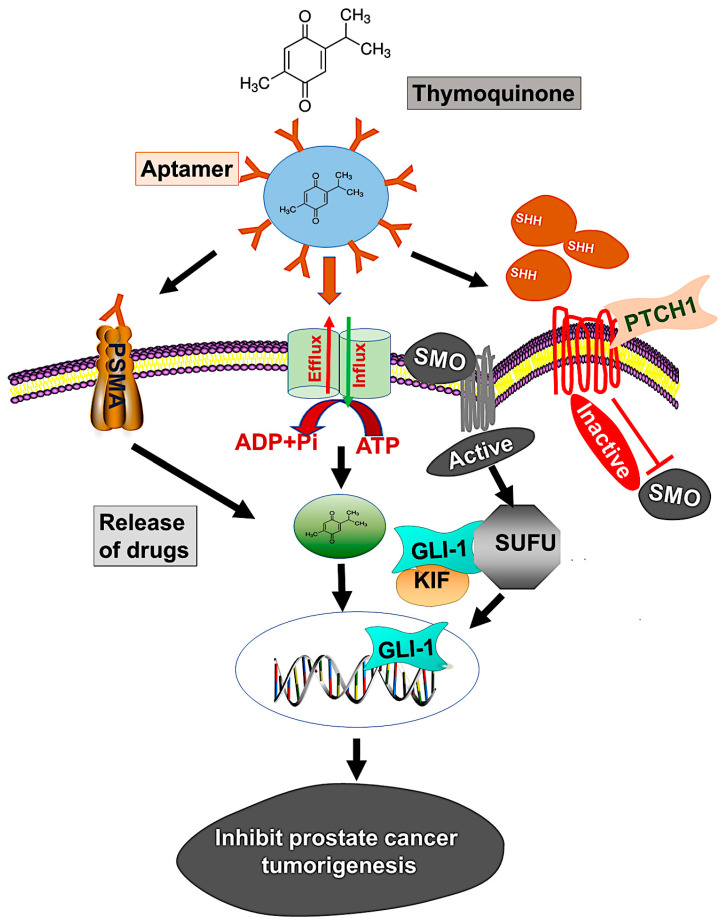
Model illustrates targeting of the hedgehog (Hh) signaling using PSMA aptamer conjugated PBM-NPs inhibits prostate cancer tumorigenesis. The Hh pathways induces chemoresistance through regulating the transcription of ABC transporter gene. Moreover, the Hh pathway include the activation of SHH which bind to the receptor PTCH1, followed by inhibition of the seven-transmembrane protein smoothened (SMO) and subsequently triggering GLI-dependent transcription. A GLI activity is regulated by loss of function mutation in suppressor of fused (SUFU), which binds to all three GLIs (GLI-1, GLI-2, and GLI-3) and controls the expression of downstream proteins. We demonstrated the mechanism by which thymoquinone (TQ) encapsulated A10 aptamer-conjugated PBM-NPs (A10-TQ-PBM-NPs) specifically bind to the PSMA receptor, expressed on cell surface. The aptamer-conjugated nanoparticles were efficiently internalized through the receptor via endocytosis and inhibited thee ABC drug efflux protein (MDR1). The PBM-NPs binds to SHH protein, thereby arresting cell proliferation and affecting other properties of PCa cells.

Although various techniques are available for treating localized or advanced cancer, the NP drug delivery system is expected to improve current strategies for treatment. However, rapid clearance of NPs from the reticuloendothelial system, biodistribution, blood circulation half-life, stealth properties, and tissue-specific accumulation are issues for effective drug delivery [58]. To overcome these barriers, our PBM-NP was fabricated with biodegradable PEG-PCL, which extends its time in circulation. In addition, for the delivery of PBM-NPs, we target PSMA, a receptor that is highly expressed on androgen-independent PCa cells. To target PSMA, a specific RNA aptamer, A10-3.2, was encapsulated in PBM-NPs loaded with TQ. Our results from the SPR analysis displayed, A10-PBM-NP has a strong interaction with the PSMA receptor. Furthermore, targeted PBM-NPs may not only release the optimal therapeutic dose, but may also overcome biological barriers. Moreover, physicochemical properties such as size, surface charge, and functional group affect the cellular uptake and clearance of NPs [58]. Our targeted PBM-NP is negatively charged, and has a small particle size and high binding capacity to the positively charged membrane [28]. In view of the established advantages of using aptamers along with NPs, we present here a study of the targeted delivery of an anti-cancer agent, TQ, which was efficacious in low concentrations compared to TQ bound to NPs. A model by which to target the hedgehog (Hh) signaling pathway using a PSMA aptamer (A10) conjugated TQ-PBM-NPs that inhibits prostate cancer tumorigenesis is shown in Figure 8.

## 5. Conclusions

In conclusion, our finding that TQ inhibits Hh signaling provides the perspective that natural anti-cancer compounds can be safer, more available, and more affordable for treating cancers driven by Hh signaling. In the present study, we found that aptamer-based NPs carrying a natural drug (TQ) allowed a reduced drug concentration, binding to a specific target, and the delivery of the agent to the target cells. Along with demonstrating that TQ controls altered the expression of the Hh pathway molecules in PCa, we provide evidence that drug resistance and the Hh pathway are interlinked and that TQ reverses the overexpression of ABC transporter genes. In summary, we have devised a new strategy involving the use of aptamer-bound anti-cancer NPs for the treatment of cancers controlled by the Hh pathway.

## Figures and Tables

**Table 1:** List of primer sequences.

	Forward	Reverse
18S	GGCCCTGTAATTGGAATGAGTC	CCAAGATCCAACTACGAGCTT
ABCB1	TGACATTTATTCAAAGTTAAAAGCA	TAGACACTTTATGCAAACATTTCAA
PSMA	GCTTCCTCTTCGGGTGGTTT	TCCTGCTAAATGTGGTATCTGTGT
SHH	TCT CCAGAAACTCCGAGCGA	ACTTGTCCTTACACCTCTGAGTC
SMO	GCTACAACGTGTGCCTGGG	CATTCCGGAGGCCCGAC
PTCH1	TGAAATCCAAGCCCAGCGTC	CAGTAGCCTTCCCCATAGCC
PTCH2	CCGCCAGAGGTGATACAGAT	CCACGGTCATGGAGGTAGTC
GLI1	GCTGCCGTGGCCCTC	GTGTGGGGACACTCTGTCTG
GLI2	ACTTGATGTTCCCTGCGCTC	CTGCGGCACCAGCGT
GLI3	GCTCCACGACCACTGAAA AG	TGTCCAGGACTTTCATCCTCATTA
SUFU	CCACACCTGCAAGAGAGAGT	TTGGCACTGACACCACTCAG
KIF	GCGCCGCACTGGGGAT	CTCTGGGCCCTGCCTGG
CK1	TCTTCAAGTGGGCAGGGTCA	CTGCTCCGATCATCTCGTCT

## Supplementary Materials

The following are available online at https://www.mdpi.com/2073-4409/9/9/1976/s1. The cell viability assay (MTT) data are attached in Supplementary Figure S1, Figure S2: Densitometry readings/intensity ratio of ABCB1 marker in C4-2B-R and LNCaP-R immunoblots, Figure S3: Densitometry readings/intensity ratio of hedgehog pathways in C4-2B-R and LNCaP-R immunoblots. Figure S4: Densitometry reading/intensity ratio of PSMA protein in PCa cells.
