# Supplementary material for: Selective Targeting of the Hedgehog Signaling Pathway by PBM Nanoparticles in Docetaxel-Resistant Prostate Cancer
Source: Cells. 2020 Aug 27;9(9):1976. doi: 10.3390/cells9091976 (PMC7563377; doi:10.3390/cells9091976)
Supplement: Supplementary file 1 [file cells-09-01976-s001.pdf]

Supplementary Information

Selective targeting of the hedgehog signaling pathway by PBM nanoparticles in docetaxel-resistant prostate cancer

Santosh Kumar Singh [1], Jennifer B. Gordetsky [2], Sejong Bae [3], Edward P. Acosta [4], James W. Lillard Jr. [1], and Rajesh Singh [1] *

Affiliations:

[1] Department of Microbiology, Biochemistry and Immunology, Cancer Health Equity Institute, Morehouse School of Medicine, Atlanta, GA, USA, 30310

[2] Departments of Pathology and Urology, Vanderbilt University Medical Center, Nashville, TN USA, 37232

[3] Division of Preventive Medicine, University of Alabama at Birmingham School of medicine, Birmingham, AL, USA, 35205

[4] Department of Pharmacology and Toxicology, University of Alabama at Birmingham, Birmingham, AL, USA, 35294

* **Corresponding author:** Rajesh Singh, Ph.D., Department of Microbiology, Biochemistry and Immunology, Cancer Health Equity Institute, Morehouse School of Medicine, 720 Westview Drive SW, Atlanta GA 30310, Tel: 404-756-6661, Fax: 404-752-1179, E-mail: rsingh@msm.edu

Supplementary Figure

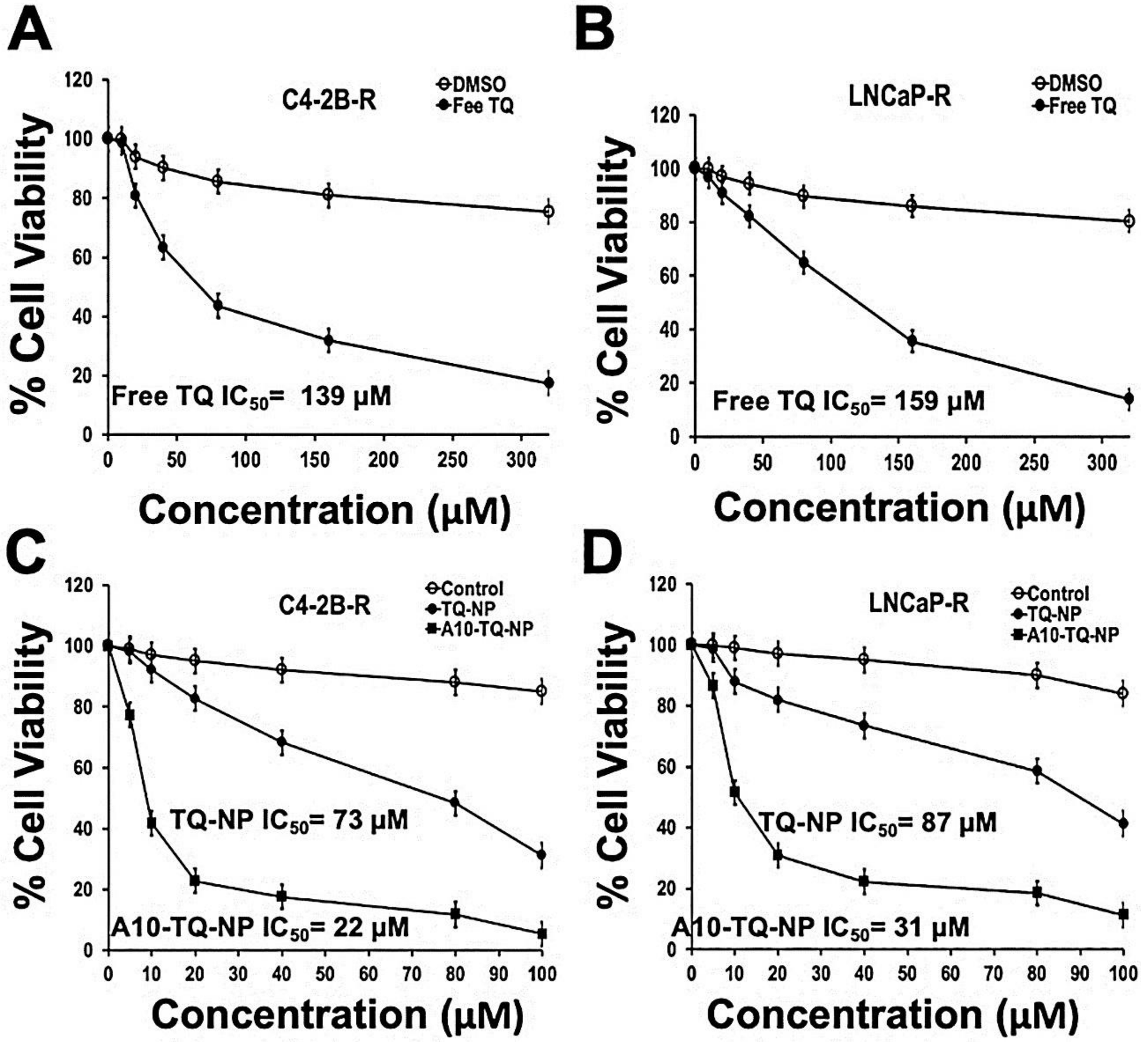

Figure S1: Thymoquinone induces an anti-proliferative effect on docetaxel resistance to prostate cancer cells. Docetaxel resistance cells, C4-2B-R and LNCaP-R were grown on 96-well plates for overnight and then treated with sequential dosages of free TQ (10, 20, 40, 80, 160, 320 µM), and PBM-NPs (TQ-NP, or A10-TQ-NPs; 5, 10, 20, 40, 80, 100 µM) for 48 h at 37°C in a 5% CO_2 incubator. Next, the cell viability was measured by MTT assay. The optimal IC_{50} values of free TQ, and PBM- NPs (TQ-NP, or A10-TQ-NP) are shown in graphs A, B, C, and D, respectively. Data are represented as a mean ± standard error of the mean of at least three independent experiments.

Figure S2. Densitometry readings/intensity ratios in C4-2B-R and LNCaP-R immunoblots

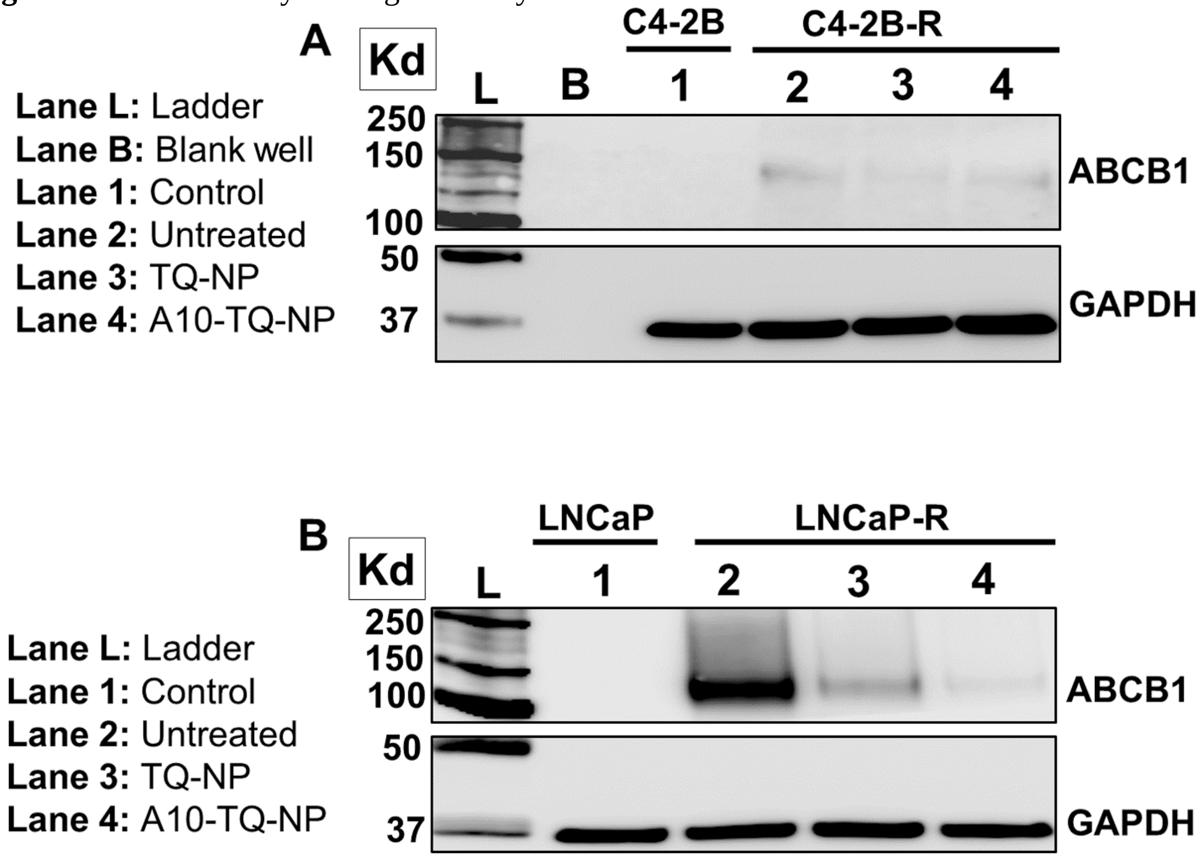

Table. Densitometry readings/intensity ratios

	**ABCB1/GAPDH**
**C4-2B-Control**	0
**C4-2B-R (UT)**	3.317917
**TQ-NP**	0.59683
**A10-TQ-NP**	0.560444

	**ABCB1/GAPDH**
**LNCaP-Control**	0
**LNCaP-R (UT)**	3.913951
**TQ-NP**	0.695781
**A10-TQ-NP**	0.137985

Figure S2. Densitometry readings/intensity ratio of ABCB1 marker in C4-2B-R and LNCaP-R immunoblots. PCa cells (parental control and DTX- resistant cell) were treated with or without A10-conjugated TQ-NPs for 48 h, and ABCB1 (~140KD) protein expression was assessed by Western blots. Tables shows the density intensity ratio of C4-2B-R and LNCaP-R cell lines. GAPDH (37KD) was used as an internal standard for equalizing the samples. The ratios for immunoblot densitometry readings/intensity shown are representative of three independent experiments.

Figure S3. Densitometry readings/intensity ratios in C4-2B-R cell line

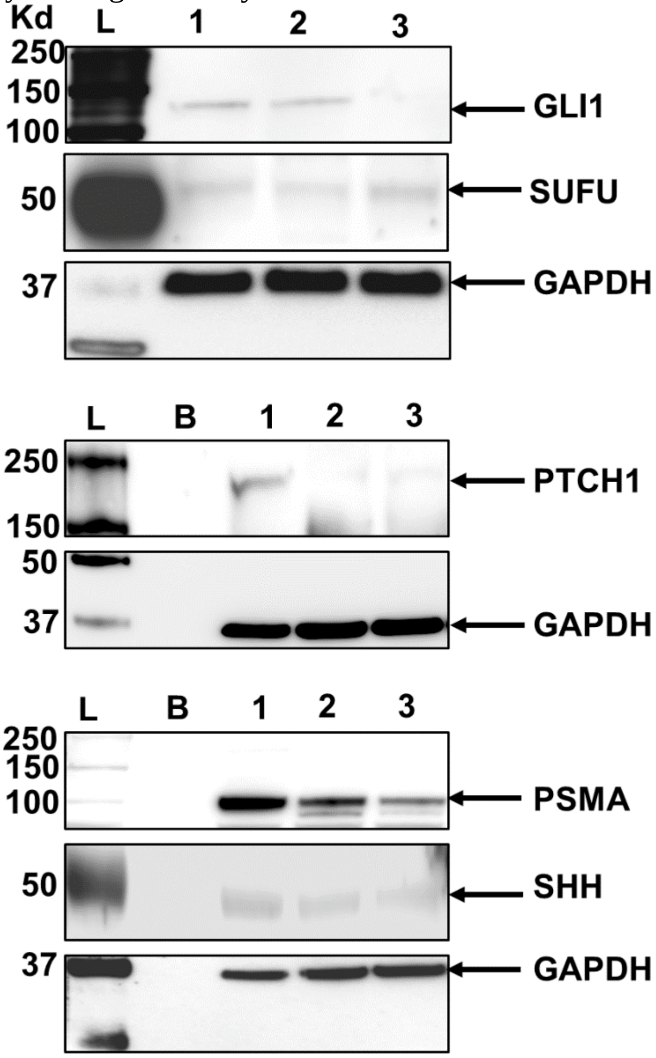

Lane L: Ladder
Lane B: Blank well
Lane 1: Untreated
Lane 2: TQ-NP
Lane 3: A10-TQ-NP

C4-2B-R					
	**PSMA/GAPDH**	**SHH/GAPDH**	**PTCH1/GAPDH**	**GLI1/GAPDH**	**SUFU/GAPDH**
**UT**	2.116074	1.550368	2.396878	1.586341	0.941512
**TQ-NP**	0.723468	0.809105	0.349075	1.368609	0.747718
**A10-TQ-NP**	0.137971	0.636629	0.398266	0.06786	1.439391

Figure S3. Densitometry readings/intensity ratios in LNCaP-R cell line

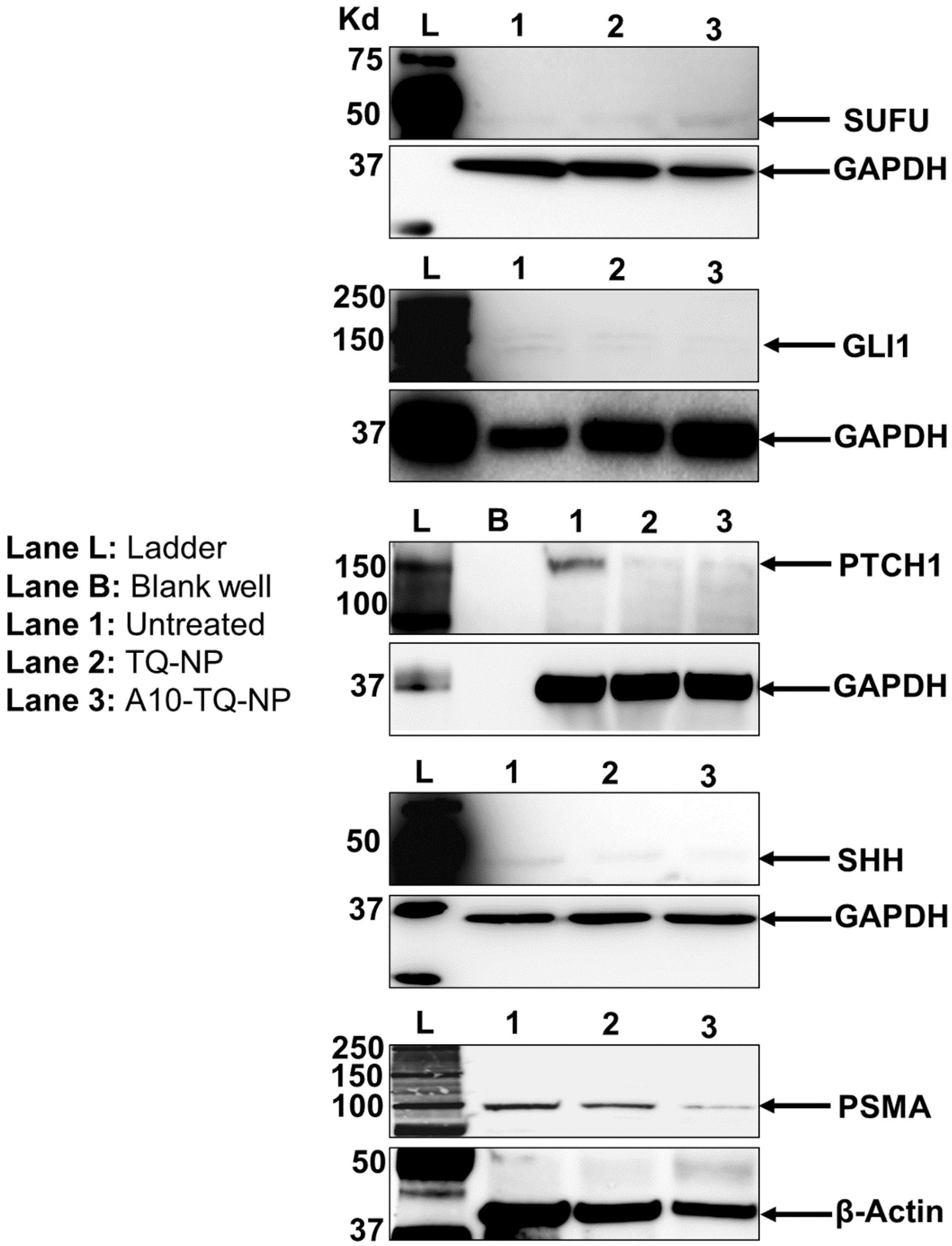

LNCaP-R					
	**PSMA/β-Actin**	**SHH/GAPDH**	**PTCH1/GAPDH**	**GLI1/GAPDH**	**SUFU/GAPDH**
**UT**	1.198227	0.921141	1.234737	0.993969	0.126035
**TQ-NP**	1.071506	0.54844	0.290596	0.728331	0.44728
**A10-TQ-NP**	0.357167	0.055435	0.258125	0.344001	2.498563

Figure S3. Densitometry readings/intensity ratio of hedgehog pathways in C4-2B-R and LNCaP-R immunoblots. DTX-resistant (C4-2B-R and LNCaP-R) PCa cells were treated with or without A10-conjugated TQ-NP for 48 h, and the expression of PSMA(110KD), SHH (45Kd precursor), PTCH1 (180KD), GLI1 (160KD), and SUFU (54KD) proteins were analyzed by Western blots. Table shows the corresponding densitometry reading intensity ratio in C4-2B-R and LNCaP-R cells. GAPDH (37KD) and β-actin (42KD) was used as a loading control. UT: untreated (DTX resistant). The ratios for immunoblot densitometry readings/intensity shown are representative of three independent experiments.

Figure S4. Densitometry readings/intensity ratios of PSMA in PCa cell lines

A

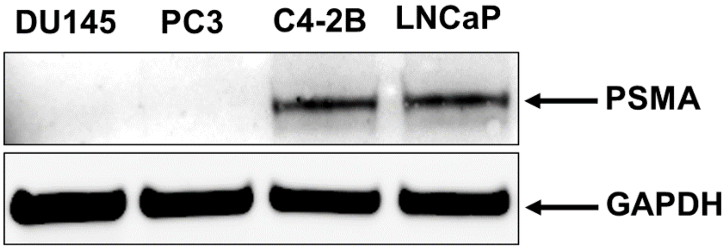

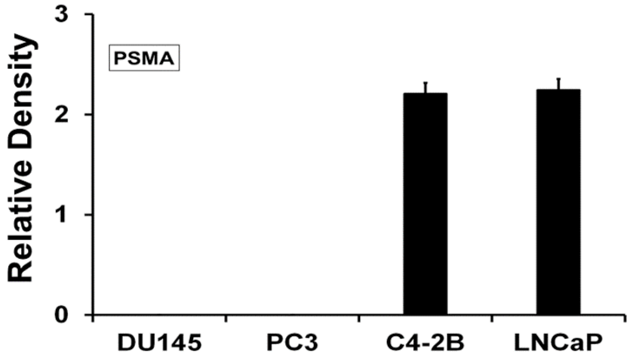

	PSMA/GAPDH
**DU145**	0
**PC3**	0
**C4-2B**	2.204945
**LNCaP**	2.24207

B

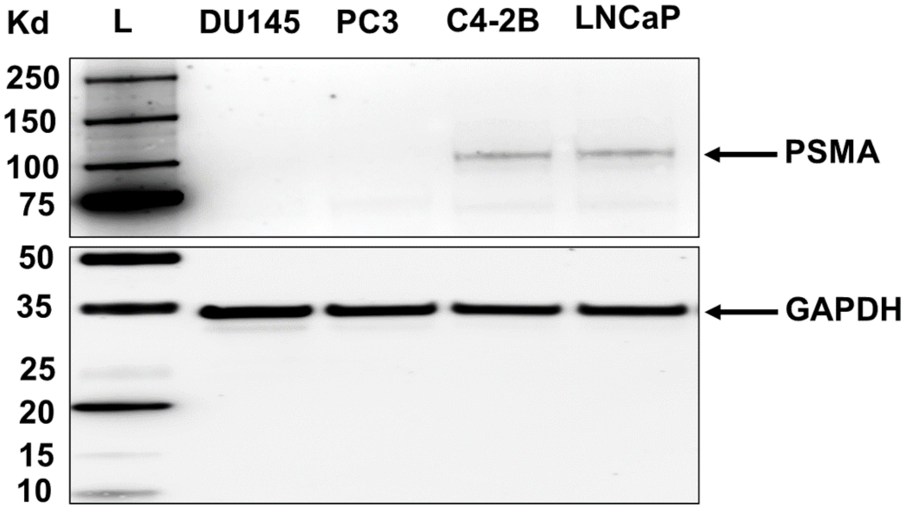

Figure S4. **Densitometry readings/intensity ratio and immunoblots of PSMA expressing PCa cell lines.** (A) Immunoblots shows the expression of PSMA (110KD) protein in untreated PCa cells (DU145, PC3, C4-2B, and LNCaP). Table shows the corresponding densitometry reading intensity ratio. GAPDH (37KD) was used as a loading control. The ratios for immunoblot densitometry readings/intensity shown are representative of three independentexperiments. PCa cell lines, PC-3 and DU145, were purchased from American Tissue Culture Collection (ATCC, Manassas, VA, USA) and maintained in RPMI-1640 media supplemented with 10% FBS, nonessential amino acids, HEPES, 2 mM L-glutamine, and penicillin/streptomycin antibiotic solution (Fisher Scientific, USA). (B) Shows the original blot of PSMA and GAPDH proteins expression.
